# Rapid Extrication of entrapped victims of motor vehicle accidents; a feasibility study

**DOI:** 10.1186/1757-7241-21-S1-S17

**Published:** 2013-05-28

**Authors:** AS Johnsen, S Fattah, JE Andersen, T Vigerust, M Rehn

**Affiliations:** 1 Department of Research and Development, Norwegian Air Ambulance Foundation, Norway; 2 Department of Emergency Medicine, Ullevål, Oslo University Hospital, Norway; 3 Anaesthesia and Critical Care Research Group, University of Tromsø, Norway; 4 Department of Anaesthesia and Intensive Care, Akershus University Hospital, Norway

## Background

Prompt and safe extrication of motor vehicle accident patients is essential to allow efficient transport to hospital. A rapid extrication technique was developed in the Oslo-Akershus Emergency Medical Services (EMS) and fire service in 1999. The Norwegian Air Ambulance Foundation trains fire fighters, police and EMS personnel in this technique. This study investigated how well the technique can be learned by rescue personnel and the extent of its implementation. A previous study indicates that rapid extrication is an a more efficient alternative than previously existing techniques [1].

## Method

Extrication times by teams interested and trained in the method were recorded during the Norwegian National Championship in Rapid Extrication. A questionnaire study was conducted after the contest. Answers were given on a Lickert scale ranging from one to seven. A cross-sectional study to investigate to which extent fire-fighting services have implemented the method is on going.

## Results

The mean time from start to end of exercise was 13 minutes 56 seconds (range: 12 minutes 25 seconds to 19 minutes). They trained the technique in teams on average 2,7 times a year (range 0-4). Self-reported security of crew scored 6,7 (range 4-7), patient safety 6,7 (5-7), communication between personnel 6,6 (3-7), teamwork 6,7 (5-7), and how well the technique functioned 6,7 (5-7).

## Discussion and conclusion

Participants were satisfied with security, communication, teamwork and how the technique functioned. Time expenditure was good; all teams had the patient in the ambulance within 20 minutes. These are critical factors to prevent sustained hypoxia, uncontrolled bleeding, hypothermia and for overall survival of the seriously injured trauma patient.
